# Reasons and Determinants of BoNT-A Treatment Discontinuation in Patients Living with Spasticity: A 10-Year Retrospective Analysis

**DOI:** 10.3390/toxins14100675

**Published:** 2022-09-29

**Authors:** Nicoletta Cinone, Luigi Santoro, Stefania Spina, Salvatore Facciorusso, Marco Battaglia, Alessio Baricich, Pasqua Marcogiuseppe, Andrea Santamato

**Affiliations:** 1 Spasticity and Movement Disorder Unit, Physical Medicine and Rehabilitation, Policlinico Riuniti, University of Foggia, Viale Pinto 1, 71122 Foggia, Italy; 2 Villa Beretta Rehabilitation Center, Valduce Hospital, Via Nazario Sauro 17, 23845 Costa Masnaga, Italy; 3 Physical Medicine and Rehabilitation, Department of Health Sciences, Università del Piemonte Orientale, viale Piazza d’armi 1, 28100 Novara, Italy

**Keywords:** botulinum toxin, spasticity, discontinuation

## Abstract

Background: The present study aimed to evaluate the reasons and determinants of BoNT-A discontinuation in patients with stroke, multiple sclerosis, spinal cord injury, and traumatic brain injury. Methods: It is a retrospective study of 56 discontinuer patients treated with botulinum toxin between January 2011 and December 2021. Discontinuation rates and their predictors were estimated using Kaplan–Meier, Log rank test, and Cox’s regression method of analyses. Results: The mean age was 56.54 years, 53.57% were affected by post-stroke spasticity, 17.86% by spinal cord injury, 12.5% and 16.07% by traumatic brain injury and multiple sclerosis, respectively. The median discontinuation time was 5 months. The main reason for discontinuation were logistic problems (37%) and orthopedic surgeries or intrathecal baclofen (27%). Discontinuers were more likely to have severe spasticity (R = 1.785), have no pain (HR = 1.320), no access to rehabilitation services (HR = 1.402), and have cognitive impairment (HR = 1.403). Conclusions: The main reasons for discontinuation are related to logistic issues (due to distance or the absence of an adequate caregiver) and surgical interventions for spasticity, including intrathecal baclofen. It is crucial to identify possible predictors of discontinuation to improve the effectiveness of a multidisciplinary management. The study confirms the crucial role of rehabilitation and caregivers in achieving better long-term outcomes.

## 1. Introduction

Spasticity is a well-known motor dysfunction arising from upper motor neuron lesions due to stroke, spinal cord injury, multiple sclerosis, and traumatic brain injury. Estimates of the incidence and prevalence of spasticity vary due to the lack of a strict definition and clinical measurement of spasticity [1]. The rate of post-stroke spasticity has been reported to be 4 to 27% during the first six weeks after onset, 19% at three months, 21.7 to 42.6% at four and six months, and 17 to 38% at 12 months. It is estimated to occur in around 80% of persons with multiple sclerosis [2] and 65–78% in those with spinal cord injuries [3]. Spasticity in people with traumatic brain injuries (TBI) depends on the severity of the injury andthe prevalence is up to 40% with severe brainstem involvement [4].

Long-term consequences of spasticity include pain, distorted joint position, posture and hygiene difficulties, and in the long term, permanent joint contractures and deformities [5,6,7,8].

Despite the ease of diagnosis, effective spasticity management is often challenging for clinicians. The goals of spasticity management may include increasing mobility and range of motion, attaining better hygiene, improving body image and functional level, and facilitating splint wear [9,10,11,12,13]. 

Botulinum toxin type A (BoNT-A) is a well-established treatment for treating focal spasticity in the upper motor neuron syndrome [14]. Over the past 30 years, accumulated evidence has established the effectiveness of BoNT-A [11,15,16,17]. BoNT-A plays also a role in the management of other conditions, such as chronic migraine, focal dystonia, hemifacial spasm, myofascial pain, and cerebral palsy [18,19,20,21]. Despite the considerable and well-known beneficial effects, many patients suffering from spasticity discontinue treatment. Consequently, there is a lack of knowledge about BoNT-A discontinuation and its determinants in daily clinical practice. 

In a previous study, Latino and coll. retrospectively collected data on patients with multiple sclerosis treated with BoNT-A from 2002 to 2014 and analyzed the reasons for treatment discontinuation [22]. No other studies to date, have been conducted so far to investigate the long-term effectiveness and persistence of treatment with BoNT-A in patients suffering from spasticity in stroke, traumatic brain injury (TBI), spinal cord injury (SCI), and multiple sclerosis (MS). Important aspects of discontinuation of BoNT-A include both the reasons for discontinuation and treatment characteristics that are associated with a higher incidence of discontinuation. The results may be useful to differentiate patient-driven discontinuation from caregiver difficulty since spasticity is a complex health condition in which the patient is only partially involved.

The present study aimed to determine the rate of treatment discontinuation over 10 years in Italian patients living with spasticity deriving from stroke, MS, SCI, and TBI.

The primary outcome was the main reason for discontinuation; secondly, we focused our attention on the durability of the treatment, defined as the time in months from the beginning of the first BoNT-A regimen until its discontinuation for any cause and the potential determinants of discontinuation.

## 2. Results

### 2.1. Patients Sociodemographic Characteristics:

In the past decade, 75 patients have discontinued BoNT-A treatment. Excluding the deceased patients, 62 patients met the inclusion criteria, while six were not contactable. Finally, a total of 56 discontinuers (Females: *n* = 26, Males: *n* = 30) satisfied the inclusion criteria and were included in this analysis. Table 1 summarizes the sociodemographic characteristics. The mean age is 56.54 years with a standard deviation of 18.73 (range 18–88 years). Most of the patients were over 60 (*n* = 30), and there were only five patients under 30 (8.93%).

### 2.2. Patients’ Clinical Characteristics

The commonest etiology was stroke [30, (53.57%)], followed by spinal cord injury [10, (17.86%)], multiple sclerosis, and traumatic brain injury accounted respectively 16.07% and 12.5% (Table 1). 

**Table 1:** Sociodemographic characteristics of discontinuers.

Variables	Discontinuers
No of patients	*n* = 56
Age (Mean)	56.54 (18.73)
Sex n (%)	-
Male	30 (53.57%)
Female	26 (46.43%)
Variable	Frequency %
Age group distribution	5 (8.93%)
≤30	8 (14.29%)
31–40	6 (10.71%)
41–50	6 (10.71%)
51–60	13 (23.21%)
>70	18 (32.14%)
Disease	-
Stroke	30 (53.57%)
Multiple Sclerosis	9 (16.07%)
SCI	10 (17.86%)
TBI	7 (12.5%)
Neurological picture	-
Monoparesis	0
Hemiparesis	32 (57.14%)
Paraparesis	24 (42.86%)
Tetraparesis	9 (25%)
Severity of UL spasticity	-
Mild	15 (26.79%)
Moderate	24 (42.86%)
Severe	17 (30.36%)
Severity of LL spasticity	-
Mild	12 (21.43%)
Moderate	14 (25%)
Severe	30 (53.57%)
Functional status	-
No disability	9 (16.07%)
Moderate disability	18 (32.14%)
Severe disability	29 (51.79%)
Comorbidities	-
None	3 (5.36%)
Moderate	16 (28.57%)
Severe	37 (66.07%)
Duration of spasticity	-
<3 years	21 (37.5%)
3–5 years	5 (8.93%)
5–10years	7 (12.5%)
>10 years	23 (41.07%)
Pain	-
Yes	12 (21.43%)
No	44 (78.57%)

UL upper limb; LL lower limb. Data are reported as frequency (%).

Hemiparesis, paraparesis, and tetraparesis accounted for 32 (57.14%), 24 (42.86%), and 9 (25%), respectively. The severity of the overall spasticity was interpreted based on clinical experience as mild, moderate, or severe related to the last injection visit. Functional status was measured using the Barthel Index, with 100 points indicating no disability, 60–95 points indicating moderate disability and 0–55 points indicating severe disability [23]. Comorbidities were measured using the Charlson Comorbidity Index, with 0 point indicating none, 1–2 points indicating moderate comorbidities and ≥3 points indicating severe comorbidities [24]. The majority of patients, presented spasticity from less than 3 years (37.5%) and over 10 years (41.07%).

### 2.3. Reasons for Discontinuing BoNT-A Injection

**Figure 1:**
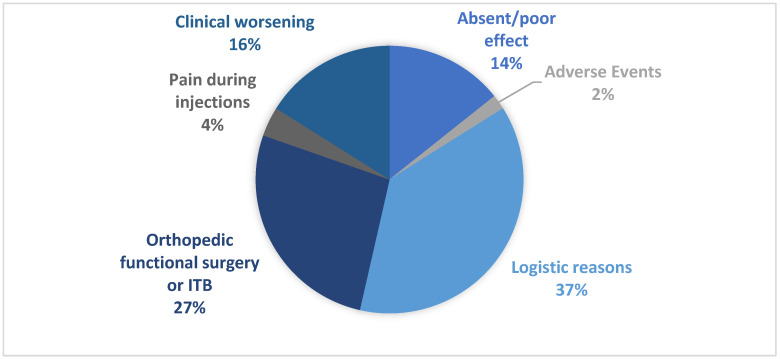
The main reason for discontinuation for patients *n* = 56.

Several key themes of factors influencing BoNT-A discontinuation were identified: unmet efficacy and adverse events; orthopedic functional surgery or intratechal baclofen; pain during injection; clinical worsening and logistical reasons. For logistical issues, we mean contingent situations that concern the inability of the patient (or caregiver) to reach the center where to receive BoNT-A treatment. The main reasons given for treatment discontinuation are shown in Figure 1. Overall, for all discontinuers, logistic reasons (including distance problems and difficulty in being accompanied) were the most common reason reported for treatment discontinuation (21, [37.5%]), especially in stroke patients (14, [46.67%]). Orthopedic surgeries and intrathecal baclofen therapy (ITB) were frequently a reason for discontinuation for spinal cord injury (7, [70%]) and traumatic brain injury (5, [71.43%]). Progression of pathology and worsening of the clinical condition was also the most common reason for discontinuation of BoNT-A in post-stroke patients (26.67%). Importantly, only 3.3% of respondents considered adverse events (AEs) as a reason for discontinuation (Table 2).

### 2.4. Factors Associated with Discontinuation of BoNT-A injection 

**Figure 2:**
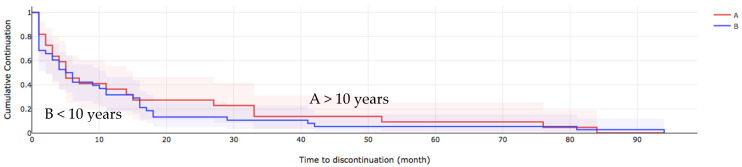
The overall survival of patients who discontinued BoNT-A treatment, spasticity duration. A > 10 years, B < 10 years.

**Figure 3:**
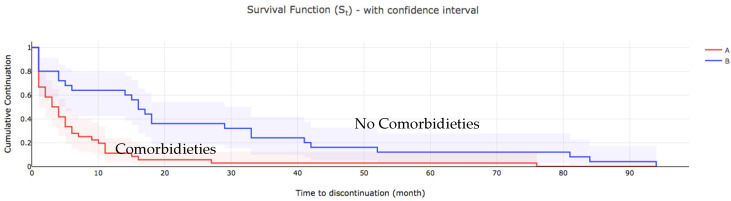
The overall survival of patients who discontinued BoNT-A treatment, comorbidities. A: comorbidities, B no comorbidities.

Table 3 shows the comparison of the estimation of the median time to discontinue care among the patients. At the bivariate analysis the severity of spasticity (*p* = 0.027), presence of comorbidities (*p* < 0.001), cognitive impairment (*p* = 0.034) presence of pain (*p* < 0.001) and access to rehabilitation (*p* < 0.001), were all associated with the median time to discontinuation of BoNT-A injections. Those who presented with severe spasticity and comorbidities had cognitive impairment with no pain were more likely to discontinue BoNT-A treatment earlier than others (Figure 2 and Figure 3). 

### 2.5. Predictors of Discontinuation of BoNT-A Injection

Table 4 showed the results of the Cox-proportional hazard model on variables associated with discontinuation of BoNT-A treatment. The multivariate analysis with the Cox-proportional hazard model showed a statistically significant association with loss to follow-up at *p* < 0.1 from Kaplan–Meier method and Log-rank test. Patients severe spasticity (95% CI = 1.047-1.639), no pain (95% CI = 1.396-2.302), with no rehabilitation regimen (95% CI = 1.120-1.760), with cognitive impairment (95% CI = 1.110-1.670) are significantly more likely to discontinue follow-up early.

## 3. Discussion

The current definition of spasticity is intended to include muscle hypertonia and other positive signs of upper motor neuron lesions, such as spasms, clonus, hyper-reflexia, and muscle coactivation [25]. To the best of our knowledge, this is the first study analyzing the reasons for BoNT-A treatment discontinuation in patients with stroke, MS, TBI, and SCI patients in the last decade. We also assessed whether BoNT-A treatment discontinuation was associated with severity of spasticity, patient characteristics, and access to rehabilitation service. 

Treatment discontinuation was defined as interruption of the initial treatment injection. Prior research indicates that discontinuance rates vary by clinical presentationin multiple sclerosis, suggesting that severalreasons may underlie patients’ premature discontinuation of treatments involved [26]. 

Discontinuance reasons are likely multifaceted (adverse events, contraindications, no effect), and the characteristics of patients who discontinue BoNT-A treatment are diverse.

Our results suggest that the main reason for discontinuation in stroke patients is related to access reasons with a crucial role of caregivers.Older age and concomitant cognitive impairments may be the main determinants.Greater disability has been shown to be associated with worse related quality of life and greater caregiver dependence [27]. Indeed, in an ASPIRE study, caregivers of patients treated with OnabotulinumtoxinA for spasticity reported less burden with a global improvement in emotional and general health, increased time spent with family and friends, more energy, and a better quality of life [28]. Although caregivers’ burden is frequently overlooked by clinicians, it has been demonstrated that caregivers are an appropriate and independent target for more focused therapeutic strategies in multiple sclerosis [28,29].

Importantly, we noticed that reduced or absent efficacy was reported in only 14% of subjects, half of them were affected by multiple sclerosis. This result matches with a previously published study, in which 37% of multiple sclerosis patients reported a loss of efficacy [18]. It is possible that prevalence figures in MS may incorrectly include motor dysfunction unresponsive to BoNT-A such as ataxia, apraxia, and fatigue [30].

The crucial balance between stiffness and paresis in MS needs attention and the risk of limiting functional autonomy should be considered. 

Similarly, 70% of patients affected by SCI and TBI reportedsurgical management of spasticity. As known, BoNT-A can be inefficacious to handle generalized spasticity; on the other hand, it has been verified that ITB treatment decreases severe generalized spasticity in patients who have predominantly lower limb spasticity. Most of the patients affected by SCI and TBI included in our study presented severe spasticity. Although the indications for BoNT-A in SCI are reduced by the generalized nature of the spasticity, there may be an indication in the incomplete forms with focal spasticity.especially in AISC and D spinal cord injuries [31]. ITB is indicated in patients with severe spasticity and spasms but more recently it has been shown to be effective in improving mobility in carefully selected individuals; prompting ITB to be considered first to improve or maintain walking. [32,33]. In the same way, ITB in ambulatory patients with multiple sclerosis was shown to reduce spasticity, spasm frequency, and self-reported pain up to 1 year post-pump insertion without negative impact on walking and transfers [34,35]. 

We observed a minor proportion of stroke patients discontinuing BoNT-A treatment for surgical management of spasticity (about 10%), although neuro-orthopedic surgery of upper limbs, and especially lower limbs, is well established [36,37]. The main reason for this is that in most cases, neuro-orthopedic surgery, which includes tendon lengthening, tendon releases, tendon transfers, and neurotomies [38], is performed at the level of the lower limb; the upper limb is still treated with BoNT-A. Overall it has to be said that BoNT-A and neuro-orthopedic surgery can coexist. Data from a systematic review show level 1 evidence that when BoNT-A is administered preoperatively, it is effective for reducing pain, spasticity, and the use of analgesics in pediatric patients with cerebral palsy. This is supported by the findings of a retrospective case series in which patients who were treated with BoNT-A injections 23–31 days before surgery had reduced post-operative pain compared with those who were not injected [39]. Level 5 evidence from case reports confirms the potential for the use of BoNT-A in the peri-operative period [40]. 

The percentage of adverse events is very low, approximately 2%. In our case, only one patient reported mild fatigue. Although randomized controlled trials could contribute data on the safety of BoNT-A, they actually do not report serious or long-term adverse events of botulinum toxin. Most serious adverse events of BoNT-A come from spontaneous reporting systems and case reports in particular higher weight-adjusted doses can increase the possibility of a systemic spread causing distant-generalized weakness [41,42].

Our analysis shows that most patients (53.57%) presented severe spasticity in lower limbs and severe disability (51.79%). This finding could be explained by the major involvement of lower limbs in patients with SCI, TBI, and multiple sclerosis. 

The mean duration of spasticity before the first cycle of injections was 14.52 months (median 5). Our results are in line with a recent study published in 2020, in which adherence to BoNT-A therapy revealed that most treatment dropouts occurred within the first 8 years [43]. In particular, authors found that long-term therapy adherence was better in blepharospasm, hemifacial spasm, and cervical dystonia patients than in spasticity patients.

We found that the predictors of discontinuation among patients treated with BoNT-A include severe spasticity and absence of pain. The lack of efficacy results in a loss of interest in continuing BoNT-A treatment. Our data show that the ineffectiveness of the first BoNT-A treatment affects discontinuation rates. We strongly believe it is therefore important to discuss with patientsand caregivers the concrete objectives that can also be achieved with subsequent injection cycles, increasing total dose, or introducing adjuvant therapies.

On the contrary, pain represents an encouraging factor of therapeutic continuity. Previously published papers showed significant efficacyof BoNT-A administration in patients suffering from neuropathic pain due to postherpetic neuralgia [44,45], SCI [46,47], peripheral nerve lesion [48], diabetic neuropathy post-traumatic/postoperative neuropathies [49,50,51], carpal tunnel syndrome [52] and stroke [53,54]. BoNT-A might improve neuropathic pain through various mechanisms such as inhibiting the secretion of pain mediators (substance P, glutamate, and calcitonin gene-related protein (CGRP)) into peripheral nerve terminals, dorsal root ganglia, and spinal cord neurons. Additionally, BoNT-A might have an anti-inflammatory effect on acute injury and chronic inflammation by reducing the release of peripheral neuro-transmitters and inflammatory mediators such as CGRP, substance P, and COX-2 [55,56]. In the present analysis, patients with cognitive impairment were more likely to be lost following BoNT-A injection. As the cognitive impairment progresses, more support is needed to keep therapeutic adherence. In our cohort, another important predictor of interruption was the absence of rehabilitation (*p* < 0.005). Latino et al. observed a similar trend in patients with MS [22]. This finding is consistent with existing literature showing that the maximum benefit of BoNT-A treatment in the management of spasticity is the association with multi-modal rehabilitation [57,58,59]. The underlying mechanism is that physiotherapy in combination with BoNT-A injections can improve the overall response to toxins, likewise by enhancing its diffusion and leading to more effective uptake by the target muscles [60]. Our results reveal that it is crucial to identify possible predictors of discontinuation to improve the effectiveness of a multidisciplinary management. Several contingency factors may influence BoNT-A treatment, especially those linked to the difficulty of accompanying patients. Long-term adherence prevents contractures or joint deformities and limits systemic side effects deriving from oral anti-spasticity medications (e.g., baclofen, tizanidine, or diazepam).When used in conjunction with other rehabilitative interventions improves spasticity outcomes, reduces the burden of care, avoids additional healthcare expenditure, and improvesthe overall quality of life [61].Therefore, the clinical benefits of BoNT-A treatment outweigh the apparent high costs of this intervention, showing it to be a cost-effective treatment.

The findings of this study have to be interpreted in light of its limitations. The present study is subject to the limitations of a retrospective database analysis. Although this contributes to the high external validity of the data, it reduces the internal validity of the dataand is susceptible to multiple sources of bias for comparing variables.We did not collect information on the single formulation of BoNT-A injected in patients included in the present study. Furthermore, it is possible that rates of adherence were affected by other reasons that have been forgotten after a long time at the time of the interview. 

## 4. Conclusions

Since spasticity is one of the aspects of the global concept of disability and it is rarely isolated, monitoring the rate and the reasons for patient adherence may be beneficial to the optimal management of spasticity. Early identification of factors predicting treatment discontinuation would allow timely adjustments for better management of the treatment plan. The main reasons for discontinuation were logistic issues and alternative management of spasticity, especially in TBI and SCI patients. The BoNT-A treatment is safe and no serious adverse events were reported.

Patients with severe spasticity, no pain, with no rehabilitation regimen are significantly more likely to discontinue follow-up early. The data from this analysis highlight patient perspectives on reasons for treatment discontinuation and the factors associated. Comprehensive care, which also includes the cognitive component, and the use of adjuvant therapies is the key message beneficial to spasticity management.

## 5. Materials and Methods

### 5.1. Patients’ Selection 

This was a retrospective cohort study of patients being treated with BoNT-A and discontinued for many reasons. The patient population consisted of all patients admitted to the “Physical and Rehabilitation Medicine department” of the University of Foggia, Foggia, Italy with a diagnosis of spasticity after stroke, MS, SCI, and TBI. 

We identified patients aged ≥18 yearsoldwho had received at least one botulinum toxin injection. We included patients with all forms of spasticity, focal or not. Hospital case files with follow-up records of patients being treated with BoNT-A attended to between January 2011 and December 2021 were retrieved from our clinic medical records by two investigators. 

The reasons for patient discontinuation were investigated involving one-to-one interviews. Each interview was voice recorded and notes taken: data were collectively analyzed, to identify a set of most common themes of discontinuation. Using a structured interview with patients, or where not possible, with the caregiver, we obtained information on potential predisposing, enabling, and reinforcing factors for discontinuation. The functional status was then measured with a telephonic Barthel Index assessment [62].

Exclusion criteria were: insufficient information in the medical records to complete the database, other movement disorder pathologies (cerebral palsy, dystonia, blepharospasm, and hemifacial spasms), and death. For patients who discontinued therapy, the duration of treatment was measured as the number of months from initiation of the first BoNT-A injection to discontinuation of therapy. The study conforms to all STROBE guidelines.

### 5.2. Statistical Analysis

Reasons for discontinuation were analyzed using frequency distribution. Factors potentially associated with discontinuation of BoNT-A injection were compared using descriptive statistics. The data were carefully entered and analyzed using SPSS version 16.0. Regular checks were done to detect and correct errors.The dependent for the outcome variable was defined as the time from the first BoNT-A injection to the discontinuation of following treatments. Discontinuation of care is defined as missing three consecutive follow-up appointments. The independent variables explored were sociodemographic characteristics and clinical factors such as age, pathology, severity of spasticity, duration of disease, functional status, pain, cognitive impairment, comorbidities, and concomitant rehabilitation treatment. Frequency, percentage, mean, median, range, and standard deviation were used to summarize the sociodemographic, clinical, and treatment variables of the patients and presented using tables and graphs. Discontinuation curves were plotted using the Kaplan–Meier method and curves were compared using Log-rank test. Log-rank test was used to test for an association between the dependent variable (loss following injection) and independent variables.

The variables were considered to show significant association when the *p* value was less than 0.05. The discontinuation of patients of spasticity was compared using Log-rank test according to clinical features. The clinical features were dichotomized for ease of comparison between the clinical subgroups. Multivariate analysis was carried out using Cox- proportional hazard models to determine the predictors of discontinuation of follow-up. This was done using covariates that showed statistically significant association with follow-up discontinuation at *p* < 0.1 on bivariate analysis. In the analysis of discontinuation using Kaplan–Meier, the time of origin was taken as the time of the first botulinum toxin injection. The endpoint of the patient was follow-up discontinuation. The median discontinuation times were obtained from the Kaplan–Meier discontinuation curve.

## Figures and Tables

**Table 2:** Reasons for BoNT-A discontinuation, % frequency. MS: Multiple sclerosis. SCI: spinal cord injury. TBI: Traumatic brain injury.

Reason for BoNT-A Discontinuation	Stroke (*n* = 30)	MS (*n* = 9)	SCI (*n* = 10)	TBI (*n* = 7)
Absent/poor efficacy	2 (6.67%)	5 (55.56%)	1 (10%)	0
Adverse Events	1 (3.33%)	-	0	0
Logistic reasons	14 (46.67%)	3 (33.33%)	2 (20%)	2 (28.57%)
Orthopedic functional surgery or ITB	3 (10%)	-	7 (70%)	5 (71.43%)
Pain during injections	2 (6.67%)	-	0	0
Clinical worsening	8 (26.67%)	1 (11.11%)	0	0

ITB: Intratechal baclofen therapy.

**Table 3:** Log-rank test estimation of BoNT-A discontinuation by clinical features. MDT= median discontinuation time; *p* < 0.05.

Variable	MDT Months	Log Rank Chi-Square χ²	*p* Value
Age			
<40 years	4	0.31	0.571
>40 years	6		
Severity of spasticity			
Mild (MAS < 2)	15	6.85	0.027
Severe (MAS 3-4)	5		
Duration of spasticity			
<10 years	5.5	0.18	0.662
>10 years	5		
Comorbidities			
Yes	3.55	10.83	<0.001
No	16		
Pain			
Yes	15.5	15.75	<0.001
No	2		
Rehabilitation			
Yes	15.5	15.75	<0.001
No	2		
Cognitive Impairment			
Yes	4.5	12.24	0.034
No	14		

**Table 4:** Cox regression analysis of hazard ratios of predictors of discontinuation of BoNT-A injection. *p* value < 0.05.

Variable	HR	95%CI	*p*-Value
Severity of spasticity			
Mild (MAS (2)	1.785	1.396–2.302	<0.001
Severe (MAS 3-4)	1.00		
Pain			
Yes	1.320	1.047–1.639	0.025
No	1.00		
Rehabilitation			
Yes	1.00	1.120–1.760	0.05
No	1.402		
Comorbidities			
Yes	1.00	0.961–1.627	0.085
No	1.256		
Cognitive Impairment			
Yes	1.403	1.110–1.670	0.034
No	1.00		

## Data Availability

The data presented in this study are available on request from the corresponding author. The data are not publicly available due to privacy issue.
